# Negative binomial modeling of musculoskeletal ultrasound grayscale histograms: a three-device comparison and harmonization study

**DOI:** 10.3389/fresc.2026.1815332

**Published:** 2026-07-02

**Authors:** Tomas I. Gonzales, Katie L. Boncella, Grace Begnell, Courtney Windsor, Michael O. Harris-Love

**Affiliations:** 1 IMS Epidemiology, University of Cambridge School of Clinical Medicine, Institute of Metabolic Science, Cambridge Biomedical Campus, Cambridge, United Kingdom; 2 Muscle, Morphology, Mechanics, and Performance Laboratory, Department of Physical Medicine and Rehabilitation, School of Medicine, University of Colorado Anschutz Medical Campus, Aurora, CO, United States; 3 Department of Biomedical Engineering, University of Colorado Denver | Anschutz Medical Campus, Aurora, CO, United States; 4 Research Service, Rocky Mountain Regional VA Medical Center, Aurora, CO, United States; 5 Department of Anesthesiology, The Ohio State University Wexner Medical Center, Columbus, OH, United States; 6 Department of Biomedical Engineering, The Catholic University of America, Washington, DC, United States; 7 Skeletal Muscle Laboratory, Research Service, Washington DC Veterans Affairs Medical Center, Washington, DC, United States

**Keywords:** device harmonization, echogenicity, grayscale histogram, inter-device variability, muscle quality, musculoskeletal ultrasound, quantitative ultrasound, tissue heterogeneity

## Abstract

**Introduction:**

Quantitative musculoskeletal ultrasound enables objective assessment of muscle morphology and clinically viable estimates of tissue composition. This approach to accessible biomedical imaging shows promise for aiding practitioners in the assessment of muscle health. However, inter-device variability in grayscale image interpretation hinders the clinical utility of quantitative ultrasound. We developed conversion models to harmonize grayscale histograms of muscle tissue across three ultrasound devices.

**Methods:**

A total of 1,368 longitudinal ultrasound images were acquired from six muscle sites in older adult men (*n* = 19; age 64.3 ± 8.3 years) using three ultrasound devices on default settings. Grayscale histograms were extracted from each muscle image and modelled using zero-inflated negative binomial regression to characterize muscle tissue echogenicity (mean grayscale value, µ) and heterogeneity (dispersion parameter, *α*). Heteroskedastic linear regression was used to develop device-to-device conversion models for µ and *α*.

**Results:**

Conversion models achieved high agreement for µ (Spearman *ρ* up to 0.906, RMSLE as low as 0.097) but showed greater errors for *α* at extremes. The device possessing the widest dynamic range, the Philips EPIQ ultrasound machine, exhibited the best performance. The device with the narrowest dynamic range, the SonoSite Titan, exhibited the poorest performance. Conversion performance did not differ by muscle site.

**Conclusion:**

We have demonstrated the feasibility of developing robust conversion models to harmonize grayscale histograms across different ultrasound devices, improving the standardization and clinical utility of quantitative musculoskeletal ultrasound. This post-processing approach provides a viable pathway for harmonizing quantitative ultrasound data across devices without raw radiofrequency access.

## 1 Introduction

Musculoskeletal ultrasound is a diagnostic imaging tool with numerous advantages over other imaging modalities, including absence of ionizing radiation exposure and lower costs (1). The echogenicity (brightness) of ultrasound images reflects muscle composition, with increased echogenicity often linked to fatty infiltration (2), atrophy (3), and neuromuscular disorders (4, 5). Similarly, the heterogeneity of the grayscale intensity distribution within a muscle, often characterized by its dispersion or variance, has been associated with pathological conditions such as sarcopenia and dynapenia (6, 7). Historically, visual assessment of muscle composition from ultrasound images has been the standard approach (8–10). Visual assessment is highly subjective and potentially insensitive to subtle changes in tissue properties, prompting the development of quantitative techniques like grayscale histogram analysis (11). While grayscale histogram analysis offers a more objective assessment of musculoskeletal tissue, a major challenge remains: quantitative measures of tissue echogenicity and heterogeneity derived from histogram data often lack comparability across different ultrasound devices due to variations in manufacturer technology and image acquisition settings (12, 13). This lack of standardization hinders the broader clinical adoption of quantitative musculoskeletal ultrasound and the viable use of muscle composition estimates to aid the assessment of muscle health (14).

To address this challenge, here we develop a set of methods to facilitate the conversion of grayscale histograms of musculoskeletal ultrasound images from three distinct ultrasound devices, with the aim to harmonize measurements across these platforms. In a convenience sample of adult U.S. Veteran participants, we first acquired longitudinal ultrasound images from six muscle sites using each device. We then extracted grayscale histograms from each image and characterized their shape using generalized negative binomial regression, modeling both the mean grayscale value (µ) which reflects tissue echogenicity, and the dispersion parameter (*α*) which reflects tissue heterogeneity. After estimating µ and *α* for each device-muscle combination across all participants, we used these parameters to develop conversion models capable of transforming grayscale histograms from one device to another. The aims of this work are to: 1) characterize inter-device differences in histogram parameters using zero-inflated negative binomial regression and 2) develop and validate conversion models for harmonization to aid data interpretation.

## 2 Methods

Nineteen U.S. Veterans participated in this single-group cross-sectional study. Inclusion criteria were: 1) a male or female U.S. Veteran; and 2) between 20 and 85 years old. Exclusion criteria were: 1) upper or lower limb amputation; 2) lower-extremity joint replacement; 3) severe cognitive impairment; 4) conditions resulting in edema; and 5) inability to read, speak, or understand English. The study was approved by the Institutional Review Board of the Research & Development Service at the Washington, DC VA Medical Center. All participants provided written informed consent.

### 2.1 Anthropometrics and ultrasound scanning of muscle sites

All measurements were performed in a clinical research environment at the Washington, DC VA Medical Center. Anthropometric measures were height and weight. Ultrasound imaging was performed by a trained and experienced (two years) single operator from a skeletal muscle laboratory using three devices, (notated as d0 through d2): 1) Hitachi-Aloka Noblus (d0); 2) Philips EPIQ 7 180 Plus (d1); and 3) SonoSite Titan (d2). Each device was set to musculoskeletal mode and operated using its factory default preset with no manual adjustments to gain or time-gain compensation. The Hitachi-Aloka Noblus (d0) used default B-mode gain and time-gain compensation (TGC) settings and tissue harmonic imaging was disabled, with a wide dynamic range provided by the ultra-broadband engine processor (65–70 dB range for musculoskeletal imaging). A linear-array transducer (5–18 MHz) was used with an effective center frequency of 10–12 MHz during image acquisition with the Hitachi-Aloka Noblus. The Philips EPIQ 7 (d1) used default gain, Smart TGC, and wide dynamic range (up to 320 dB system capability) settings, with tissue harmonic imaging disabled. A linear-array transducer (2–22 MHz) was used with an effective center frequency of 8–10 MHz during image acquisition with the Philips EPIQ 7. For the SonoSite Titan (d2), overall gain, near-field gain, and far-field gain (serving as TGC) were set to the manufacturer's mid-level “neutral” position optimized for musculoskeletal imaging; Tissue harmonic imaging was available but disabled by default. A linear-array transducer (5–10 MHz) was used with an effective center frequency of 7–8 MHz during image acquisition with the SonoSite Titan. During scanning, participants were seated upright in a straight-back chair with arms relaxed, feet flat on the floor, and knees at 90 degrees. Each examination lasted approximately 45–60 min.

Longitudinal ultrasound images were acquired, as previously described (15), from six muscle sites: the upper trapezius, pectoralis major, middle deltoid, brachioradialis, rectus femoris, and tibialis anterior. These muscles were selected for their use in prior investigations (16), accessibility within the imaging window, and proximity to surface anatomical landmarks to facilitate standardized transducer positioning. Five scans were performed for each device-muscle combination. The first scan, primarily used for establishing transducer positioning and scout image review, was not used in the analysis. The four remaining scans were saved as uncompressed Digital Imaging and Communications in Medicine image files, resulting in seventy-two images per participant. During acquisition, the operator applied minimal transducer pressure and repeated scans when image quality was insufficient for visualization of the target muscle. Images were considered usable only if the target muscle and its superficial and deep fascial borders could be adequately identified for region-of-interest placement. Images with substantial artifact or inadequate visualization were not included in the analysis. No additional artifact-correction preprocessing, such as correction for acoustic shadowing or reverberation, was applied before histogram extraction.

The following conventions (15, 17) were used to standardize transducer positioning: 1) for the upper trapezius, at the midpoint of the line from the acromioclavicular joint to the sternocleidomastoid; 2) for the pectoralis major, initially at the second intercostal space and sternal border, then moved laterally until the pectoralis minor was visible; 3) for the middle deltoid, at the midpoint of the line from the acromion process to the deltoid's insertion point on the humerus; 4) for the brachioradialis, 4 cm from the anterior cubital crease with the elbow flexed at 90 degrees, forearm neutral, and thumb vertical; 5) for the rectus femoris, at the midpoint of the line from the anterior superior iliac spine to the superior pole of the patella; and 6) for the tibialis anterior, one-third of the distance on the line from the anterior aspect of the patella to the lateral malleolus.

**Figure 1:**
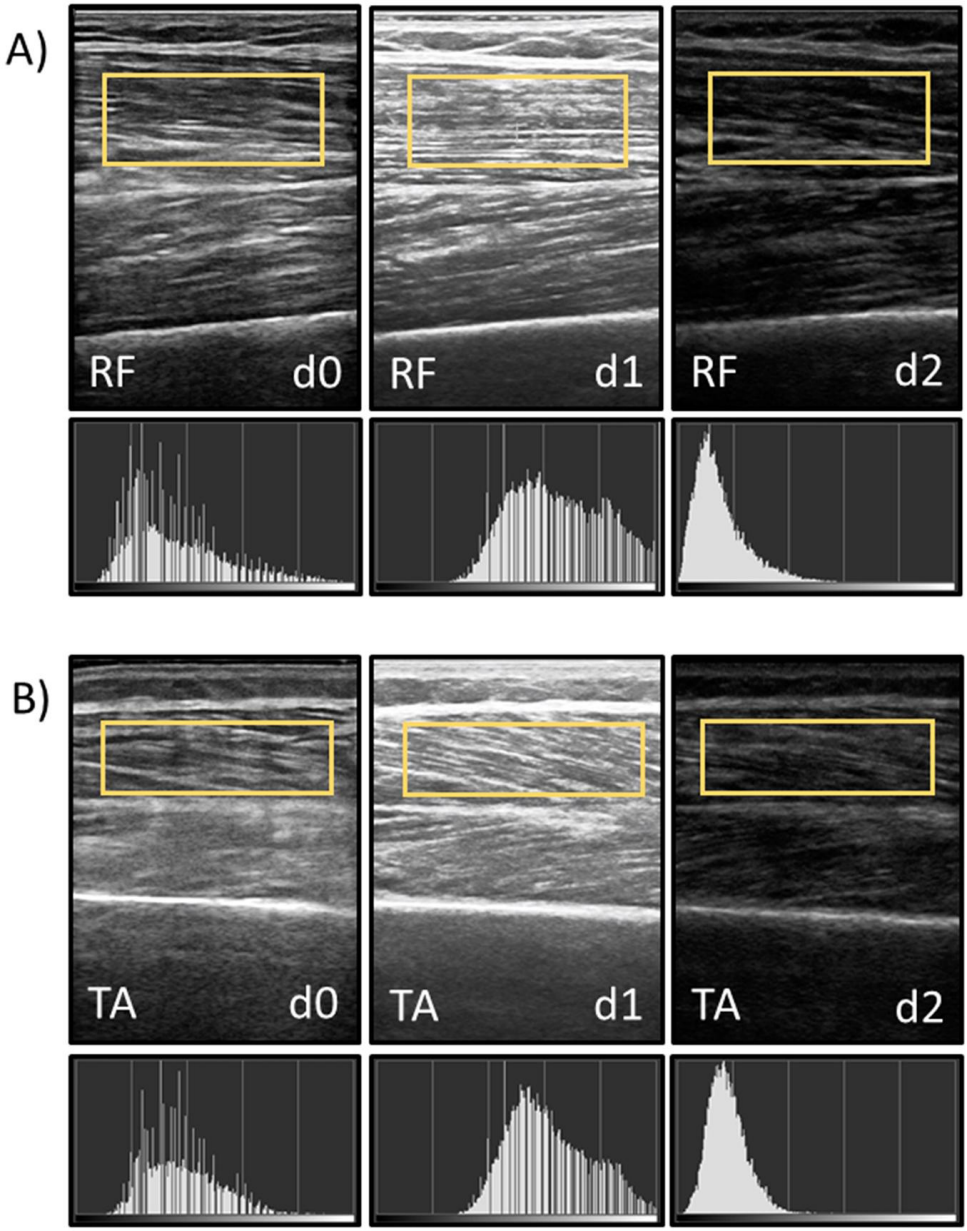
Exemplar longitudinal ultrasound images (top) and corresponding grayscale histograms (bottom) of the **(A)** rectus femoris (RF) and **(B)** tibialis anterior (TA) muscles from a single participant, acquired with three ultrasound devices (d0: hitachi-aloka noblus, d1: philips EPIQ 7 180 plus, d2: sonoSite titan). Histograms were generated from the inscribed rectangular regions of interest. Histogram x-axes represent grayscale intensity values from 0 to 255, and y-axes represent relative pixel frequency within the region of interest. B-mode images are shown as qualitative exemplars and are not presented with a common spatial scale.

Custom LabVIEW software (National Instruments Corporation, Austin, TX) was used to define a rectangular region of interest within each ultrasound image. The region of interest was manually defined to: 1) span the muscle tissue between the superficial and deep fascial borders; 2) extend laterally to include as much muscle tissue as possible; and 3) exclude extraneous structures in the field of view (e.g., subcutaneous fat, bone, other muscles). A histogram of grayscale intensity values (0–255) was computed for the region of interest, and each bin count was divided by the total number of pixels to obtain a normalized histogram. The histogram was then saved to a data file. Exemplar ultrasound images, regions of interest, and corresponding grayscale histograms are provided in Figure 1.

### 2.2 Statistical analyses of grayscale histograms and conversion model

We reshaped each image-level grayscale histogram into bin-level observations and pooled these observations across all images and participants. We then used zero-inflated generalized negative binomial regression to assess overall differences in grayscale histogram shapes across device-muscle combinations (18). Zero-inflated generalized negative binomial regression jointly models the mean grayscale value (µ) and dispersion parameter (*α*) of the distribution, accommodating overdispersion often observed in grayscale histogram data, while also modeling the probability of observing grayscale values of zero (π). Preliminary inspection of histograms from d2 (SonoSite Titan) revealed an excess of zero grayscale values, possibly due to the device's lower default gain settings truncating lower values. To account for this while maintaining a consistent modeling framework across devices, the zero-inflated model was fit across all device-muscle combinations. We characterized the overall distribution of grayscale histogram shapes across all participants by estimating µ, *α*, and π for each device-muscle combination. We then applied the same regression model to each participant's data individually to quantify individual-level variation in µ, *α*, and π across device-muscle combinations.

For grayscale histogram conversion modeling, we modeled log-transformed mean grayscale values (ln µ) and log-transformed dispersion parameters (ln *α*), rather than their raw values, to reduce scale-dependent residual variation and improve model stability. To determine the optimal set of variables to include in the conversion models, we conducted two sets of sensitivity analyses. The first sensitivity analysis was for predicting ln µ and compared an intercept-only linear regression model to models that progressively included: 1) ln *α* as a predictor; 2) muscle site as a covariate; and 3) the interaction between ln µ and ln *α*. The second sensitivity analysis was for predicting ln *α* and compared an intercept-only linear regression model to models that progressively included: 1) ln µ as a predictor; 2) muscle site as a covariate; and 3) the interaction between ln µ and ln *α*. Within each sensitivity analysis, the log-likelihood, Akaike Information Criterion (AIC), and statistical significance of the Wald test were used to assess the parsimony of each progressive model. Models were assessed for all device-to-device combinations (d0 as a predictor of d1 or d2, d1 as a predictor of d0 or d2, d2 as a predictor of d0 or d1). π was not included in the conversion models because excess zeros appeared to be device- or dataset-specific, and including π would require future applications of the conversion approach to use zero-inflated modeling even when excess zeros are not present.

Based on the results of the sensitivity analyses, we used heteroskedastic linear regression to develop the final conversion models for predicting ln µ and ln *α* across all device-to-device combinations (19). The initial log transformation was used to reduce scale-dependent residual variation in the estimated histogram parameters. Heteroskedastic linear regression was then used to model any remaining non-constant residual error directly, using separate functions for the mean outcome and residual variance. Models were fit using Stata's “hetregress” command, which models the residual variance as an exponential function of specified covariates. Variables included in the mean function were selected from the progressive modeling sensitivity analyses by considering improvement in log-likelihood, reduction in AIC, and Wald tests for newly added predictors, while prioritizing a common parsimonious model structure across all device-to-device combinations. The variance function included the same set of variables as the mean function. We additionally used cluster-robust variance estimation to account for the correlation of data from multiple images within each participant.

We used leave-one-out cross-validation (LOOCV) to assess the predictive accuracy of each device-to-device conversion model. For each participant, we withheld their data and fit the conversion models on data from the remaining participants. We then used the fitted models to predict ln µ and ln *α* for the held-out participant. This process was repeated for each participant. We then computed the root mean squared log error (RMSLE) between the predicted and observed log-transformed values across all participants. To assess agreement of raw values, we transformed the predicted values back to their original scale and calculated Spearman rank correlations between predicted and observed values. Agreement was visually inspected using scatter plots and Bland-Altman plots. Differential agreement by muscle site was examined using box plots and cluster-robust quantile regression (20).

All statistical analyses were performed using Stata (Version 17.0, StataCorp, College Station, TX). A *p*-value of 0.05 or less was considered statistically significant.

## 3 Results

A total of 1,368 longitudinal ultrasound images were acquired from six muscle sites (four images per site) in nineteen adults using three ultrasound devices. Nineteen US Veteran men were recruited for this study. No female participant candidates were identified during the course of study recruitment and enrollment. Participants tended to be older (mean ± SD age: 64.3 ± 8.3 years) and overweight or obese (mean ± SD BMI: 30.0 ± 6.8).

**Figure 2:**
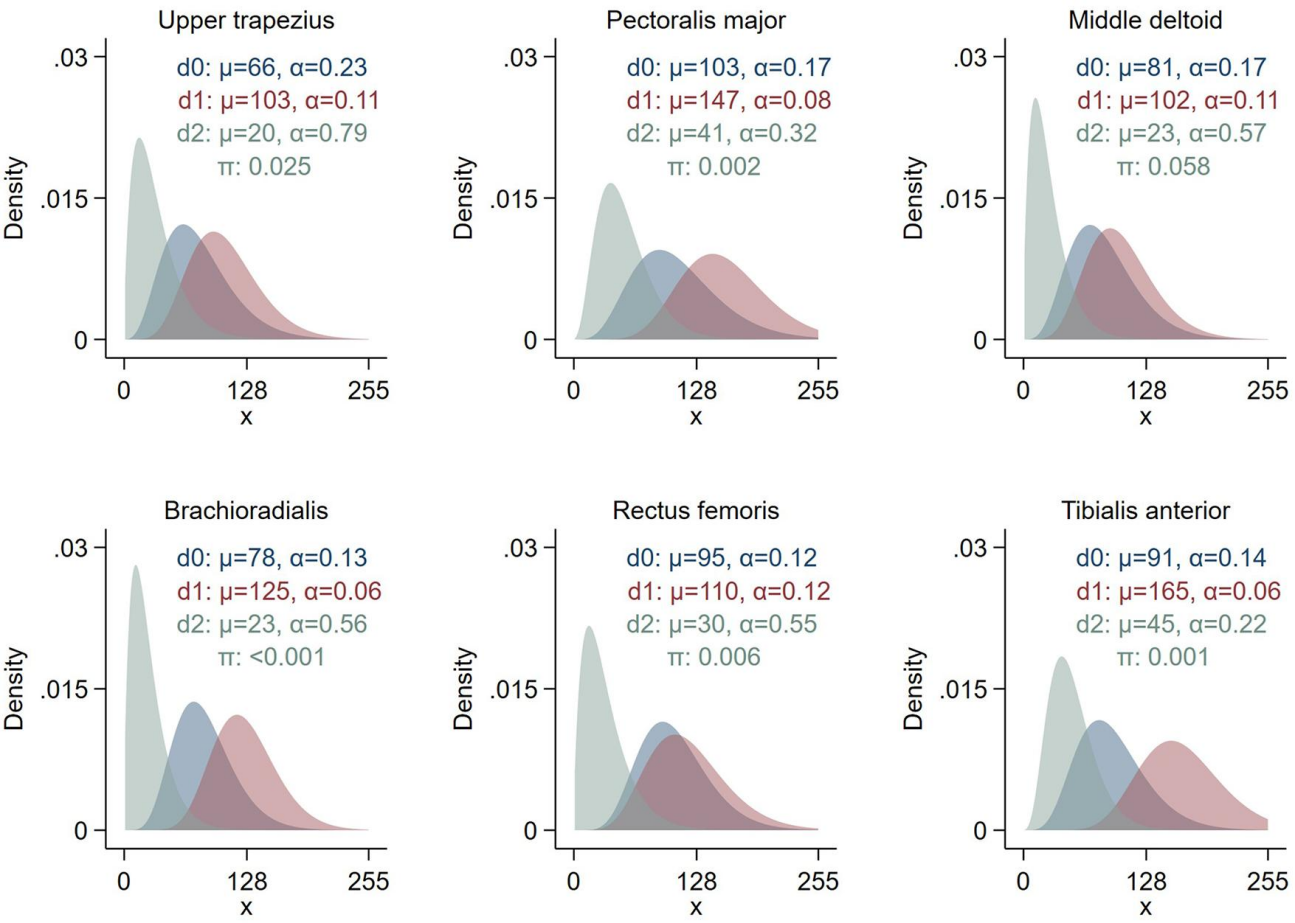
Estimated negative binomial distributions representing the overall grayscale histogram shape for each device-muscle combination (d0: hitachi-aloka noblus, d1: philips EPIQ 7 180 plus, d2: sonoSite titan). *X*-axis: Grayscale intensity range (0 to 255). *Y*-axis: Probability density (0 to 1). μ: Mean grayscale value. *α*: Dispersion parameter. π: Probability of observing grayscale values of zero. π for d1 and d2 were <0.001 for all muscle sites. π values for d2 are notated but not plotted.

Figure 2 shows estimated negative binomial distributions for each device-muscle combination, representing the overall shape of grayscale histograms across all participants. There were notable differences in grayscale histogram shapes across ultrasound devices and muscles. Across all muscles, device 2 produced images with lower echogenicity (lower µ) and greater heterogeneity (higher *α*) compared to d0 and d1. Conversely, d1 produced images with the highest echogenicity (higher µ) and lower heterogeneity (lower *α*) than d0 and d2. The relative ranking of µ and *α* values across muscles varied between devices. Overall, the tibialis anterior had the highest echogenicity and lowest heterogeneity, and the upper trapezius the lowest echogenicity and highest heterogeneity.

Table 1 presents the results of sensitivity analyses to determine the optimal device-to-device conversion model structure. For most device-to-device combinations, the optimal model structure for predicting In *μ* and ln⁡α included both In *μ* and ln⁡α as predictors, as well as muscle site as a covariate. Only a few device-to-device combinations showed a statistically significant improvement in model fit when the interaction between ln⁡α with In *μ* was additionally included (e.g., d1 as a predictor of d2), but this improvement was not observed consistently across all other device combinations. Given the overall consistency of the simpler model structure and to mitigate the risk of overfitting, we opted to use this structure in our final conversion modeling analysis.

**Table 1:** Sensitivity analysis of conversion models across device-to-device combinations using nested multivariate linear regression (in *μ*: top, ln⁡α: bottom).

Model level	Variable added	Base model	$\ln\mu_{d_{Out}}={\mathrm{Intercept}}_{\mathrm{Constant}}$					
		Outcome device	d0		d1		d2	
		Predictor device	d1	d2	d0	d2	d0	d1
		Diagnostics						
I	$\beta_{0}\cdot \ln\mu_{d_{\mathrm{Pred}}}$	LL	18.30	51.07	28.89	70.24	−28.08	−14.31
		AIC	−28.60	−94.14	−49.78	−132.48	64.16	36.63
		*p*-value	<0.001	<0.001	<0.001	<0.001	<0.001	<0.001
II	$\mathrm{Intercep}t_{\mathrm{Muscle}}$	LL	54.11	72.06	89.72	114.95	3.31	10.63
		AIC	−80.22	−116.13	−151.44	−201.90	21.37	6.74
		*p*-value	<0.001	<0.001	<0.001	<0.001	<0.001	<0.001
III	$\beta_{1}\cdot \ln\alpha_{d_{\mathrm{Pred}}}$	LL	60.79	75.50	90.86	119.74	8.66	14.42
		AIC	−89.59	−119.00	−149.72	−207.47	14.67	3.15
		*p*-value	<0.001	0.011	0.256	0.008	<0.001	0.025
IV	$\beta_{2}\cdot \ln\mu_{d_{\mathrm{Pred}}}\cdot \ln\alpha_{d_{\mathrm{Pred}}}$	LL	63.51	77.06	92.54	120.00	11.22	19.18
		AIC	−91.03	−118.11	−149.08	−204.00	13.57	−2.35
		*p*-value	<0.001	0.678	0.624	0.974	0.710	0.003

Model level	Variable added	Base model	$\ln\alpha_{d_{\mathrm{Out}}}=\mathrm{Intercep}t_{\mathrm{Constant}}$					
		Outcome device	d0		d1		d2	
		Predictor device	d1	d2	d0	d2	d0	d1
		Diagnostics						
I	$\beta_{0}\cdot \ln\alpha_{d_{\mathrm{Pred}}}$	LL	−78.91	−79.67	−43.30	−32.29	−123.89	−106.57
		AIC	165.82	167.33	94.61	72.58	255.78	221.13
		*p*-value	<0.001	<0.001	<0.001	<0.001	<0.001	<0.001
II	$\mathrm{Intercep}t_{\mathrm{Muscle}}$	LL	−62.40	−60.30	−18.30	−13.07	−79.75	−76.26
		AIC	152.79	148.60	64.59	54.15	187.50	180.53
		*p*-value	<0.001	<0.001	<0.001	<0.001	<0.001	<0.001
III	$\beta_{1}\cdot \ln\mu_{d_{\mathrm{Pred}}}$	LL	−54.82	−57.27	−17.07	3.91	−59.52	−37.80
		AIC	141.64	146.54	66.14	24.18	151.03	107.59
		*p*-value	0.030	<0.001	0.817	<0.001	<0.001	<0.001
IV	$\beta_{2}\cdot \ln\mu_{d_{\mathrm{Pred}}}\cdot \ln\alpha_{d_{\mathrm{Pred}}}$	LL	−51.25	−56.85	−16.39	7.64	−59.07	−37.44
		AIC	138.50	149.71	68.77	20.72	154.15	110.87
		*p*-value	0.222	0.424	0.246	0.250	0.806	0.861

Starting with fixed intercept-only base model, sequential models were built by adding one variable at each model level. Diagnostics were then computed at each level to assess model parsimony.

$\ln\mu_{d_{\mathrm{Out}}}$, $\ln\mu_{d_{\mathrm{Pred}}}$: Log-transformed *μ* for the outcome (*Out*) and predictor (*Pred*) devices. $\ln\alpha_{d_{\mathrm{Out}}}$, $\ln\alpha_{d_{\mathrm{Out}}}$: Log-transformed α for the outcome (*Out*) and predictor (*Pred*) devices. $\beta_{0}$, $\beta_{1}$, $\beta_{2}$: Regression model coefficients. $\mathrm{Intercep}t_{\mathrm{Constant}}$ is a fixed intercept. $\mathrm{Intercep}t_{\mathrm{Muscle}}$ varies by muscle site. LL: Log-likelihood. AIC: Akaike Information Criteria. *p*-value: Wald test for the contribution of the newly added predictor(s) to the mean function at each model level. d0: Hitachi-Aloka Noblus. d1: Philips EPIQ 7 180 Plus. d2: SonoSite Titan.

Table 2 summarizes the coefficient estimates for the final conversion models of In *μ* and ln⁡α, respectively, across device-to-device combinations. Models for predicting In *μ* generally outperformed those for predicting ln⁡α. For In *μ*, the best performing model was when d1 was the outcome device and d2 was the predictor. For ln⁡α, the best performing model was when d2 was the outcome device and d1 was the predictor. Predicting In *μ* and ln⁡α when d2 was the outcome device resulted in larger RMSE values compared to when other devices were the outcome, particularly at the extremes of the predicted values. Overall, using d1 as the outcome device resulted in the best conversion model performance.

**Table 2:** Multivariate linear regression models for predicting log-transformed mean grayscale values (in *μ*: top) and dispersion parameters (ln⁡α: bottom) for an outcome ultrasound device using in *μ* and ln⁡α from a predictor device, with muscle site as a covariate.

Prediction model	$\ln\mu_{d_{\mathrm{Out}}}=\beta_{0}\cdot \ln\mu_{d_{\mathrm{Pred}}}+\beta_{1}\cdot \ln\alpha_{d_{\mathrm{Pred}}}+\mathrm{Intercep}t_{\mathrm{Constant}}+\mathrm{Intercep}t_{\mathrm{Muscle}}$					
Outcome device	d0		d1		d2	
Predictor device	d1	d2	d0	d2	d0	d1
β coefficients						
$\beta_{0}\cdot \ln\mu_{d_{\mathrm{Pred}}}$	0.91 (0.08)	0.33 (0.05)	0.58 (0.07)	0.24 (0.04)	1.83 (0.12)	1.97 (0.15)
$\beta_{1}\cdot \ln\alpha_{d_{\mathrm{Pred}}}$	−0.16 (0.04)	−0.08 (0.03)	0.04 (0.04)	−0.06 (0.02)	0.17 (0.05)	−0.16 (0.07)
$\mathrm{Intercep}t_{\mathrm{Constant}}$	−0.41 (0.36)	3.24 (0.12)	2.36 (0.27)	3.95 (0.11)	−4.43 (0.54)	−6.65 (0.78)
$\mathrm{Intercep}t_{\mathrm{Muscle}}$						
Upper trapezius	−0.08 (0.03)	−0.09 (0.03)	−0.03 (0.02)	−0.06 (0.02)	0.07 (0.04)	−0.04 (0.05)
Pectoralis major	0.06 (0.04)	0.03 (0.03)	0.05 (0.03)	0.06 (0.02)	−0.02 (0.06)	0.06 (0.03)
Middle deltoid	0.12 (0.04)	0.08 (0.02)	−0.15 (0.03)	−0.09 (0.03)	−0.18 (0.04)	0.05 (0.05)
Brachioradialis	−0.14 (0.03)	0.01 (0.03)	0.06 (0.02)	0.07 (0.03)	−0.07 (0.06)	−0.25 (0.07)
Rectus femoris	0.27 (0.03)	0.13 (0.04)	−0.16 (0.03)	−0.09 (0.03)	−0.15 (0.05)	0.27 (0.05)
Tibialis anterior	−0.23 (0.04)	−0.16 (0.03)	0.24 (0.02)	0.11 (0.01)	0.35 (0.03)	−0.10 (0.06)
Spearman's *ρ*	0.810	0.860	0.868	0. 906	0.896	0.896
RMSE	0.149	0.127	0.117	0.097	0.243	0.243

Prediction model	$\ln\alpha_{d_{\mathrm{Out}}}=\beta_{0}\cdot \ln\alpha_{d_{\mathrm{Pred}}}+\beta_{1}\cdot \ln\mu_{d_{\mathrm{Pred}}}++\mathrm{Intercep}t_{\mathrm{Constant}}+\mathrm{Intercep}t_{\mathrm{Muscle}}$					
Outcome device	d0		d1		d2	
Predictor device	d1	d2	d0	d2	d0	d1
β coefficients						
$\beta_{0}\cdot \ln\alpha_{d_{\mathrm{Pred}}}$	0.71 (0.10)	0.68 (0.12)	0.40 (0.07)	0.65 (0.06)	0.37 (0.10)	0.94 (0.12)
$\beta_{1}\cdot \ln\mu_{d_{\mathrm{Pred}}}$	−0.71 (0.33)	0.28 (0.08)	−0.05 (0.19)	0.50 (0.09)	−1.63 (0.21)	−2.24 (0.32)
$\mathrm{Intercep}t_{\mathrm{Constant}}$	3.09 (1.53)	−2.43 (0.26)	−1.72 (0.75)	−3.69 (0.24)	6.85 (0.85)	12.24 (1.52)
$\mathrm{Intercep}t_{\mathrm{Muscle}}$						
Upper trapezius	0.09 (0.15)	0.06 (0.11)	−0.01 (0.07)	0.04 (0.04)	−0.10 (0.11)	−0.10 (0.09)
Pectoralis major	0.22 (0.09)	0.31 (0.09)	0.01 (0.07)	0.12 (0.05)	−0.11 (0.10)	−0.03 (0.08)
Middle deltoid	−0.42 (0.16)	−0.33 (0.17)	0.10 (0.06)	−0.06 (0.06)	0.38 (0.09)	−0.02 (0.11)
Brachioradialis	0.14 (0.07)	−0.09 (0.07)	−0.12 (0.07)	−0.19 (0.07)	0.10 (0.09)	0.34 (0.07)
Rectus femoris	−0.40 (0.08)	−0.35 (0.11)	0.32 (0.08)	0.08 (0.04)	0.49 (0.11)	−0.17 (0.08)
Tibialis anterior	0.37 (0.17)	0.41 (0.16)	−0.32 (0.07)	0.01 (0.05)	−0.76 (0.09)	−0.02 (0.14)
Spearman's *ρ*	0.682	0.678	0.755	0.813	0.823	0.907
RMSE	0.431	0.417	0.302	0.263	0.433	0.371

Regression model coefficients are presented as estimate (standard error). $\ln\mu_{d_{\mathrm{Out}}}$, $\ln\mu_{d_{\mathrm{Pred}}}$: Log-transformed μ for the outcome (*Out*) and predictor (*Pred*) devices. $\ln\alpha_{d_{\mathrm{Out}}}$, $\ln\alpha_{d_{\mathrm{Out}}}$: Log-transformed α for the outcome (*Out*) and predictor (*Pred*) devices. $\beta_{0}$, $\beta_{1}$, $\beta_{2}$: Regression model coefficients. $\mathrm{Intercep}t_{\mathrm{Constant}}$ is a fixed intercept. $\mathrm{Intercep}t_{\mathrm{Muscle}}$ varies by muscle site. Spearman's *ρ*: Spearman's rank correlation coefficient. RMSE: Root mean square error. d0: Hitachi-Aloka Noblus. d1: Philips EPIQ 7 180 Plus. d2: SonoSite Titan.

**Figure 3:**
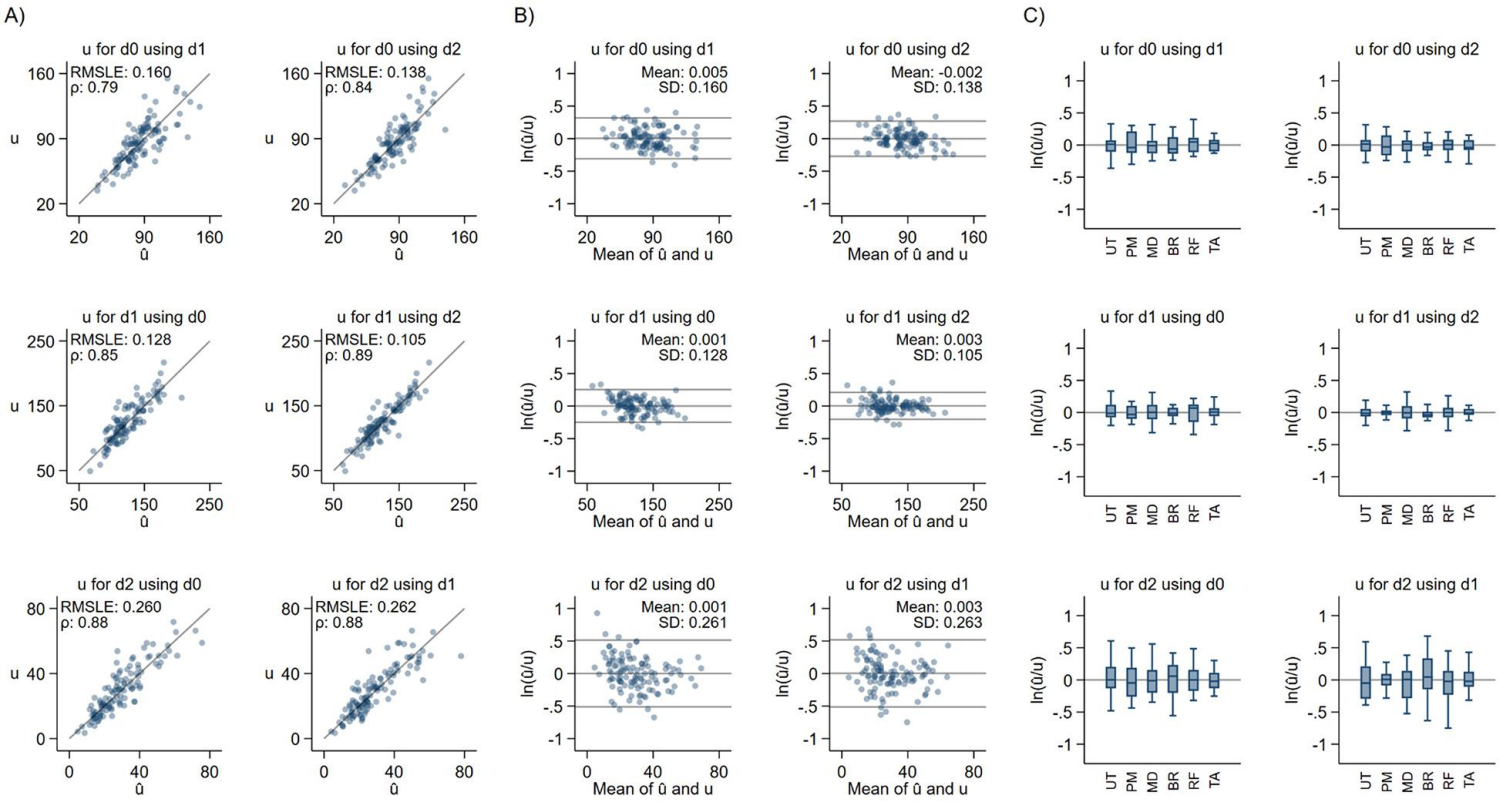
Scatterplots **(A)**, bland-altman plots **(B)**, and box plots **(C)** assessing agreement between measured mean grayscale values (μ) and predicted mean grayscale values $\left(\hat{\mu }\right)$ in leave-one-out-cross-validation (LOOCV) across device combinations (d0: hitachi-aloka noblus, d1: philips EPIQ 7 180 plus, d2: sonoSite titan). RMSLE: Root mean squared log error. *ρ*: Spearman's rank correlation coefficient. Scatter plot line is identity line. Bland-Altman plot lines are mean ± 1.96 standard deviations (SD) of $\ln(\hat{\mu }/\mu )$. Box plots demonstrate differential agreement by muscle site. UT, Upper trapezius; PM, Pectoralis major; MD, Middle deltoid; BR, Brachioradialis; RF, Rectus femoris; TA, Tibialis anterior.

**Figure 4:**
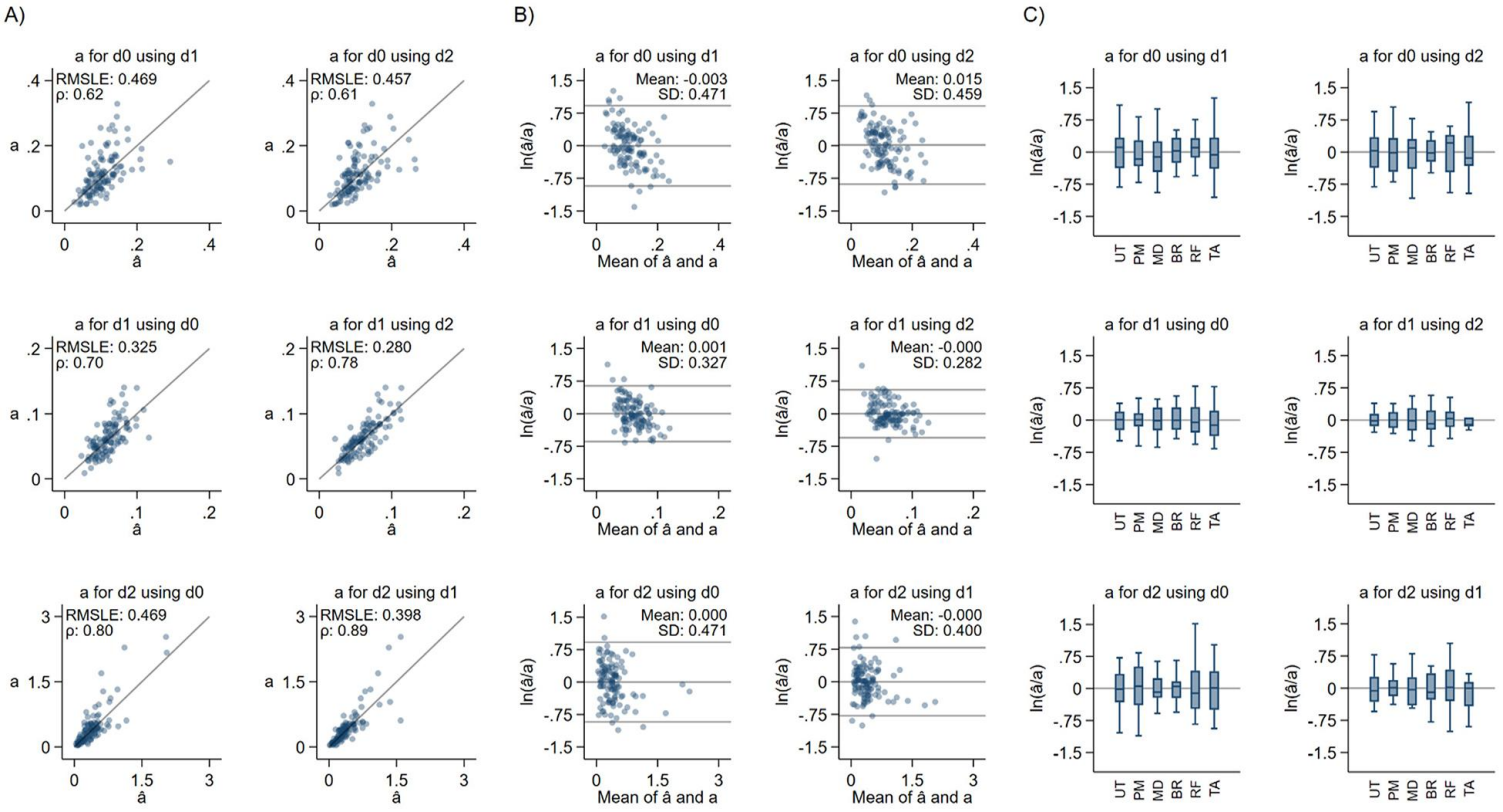
Scatterplots **(A)**, Bland-Altman plots **(B)**, and box plots **(C)** assessing agreement between measured dispersion parameters (α) and predicted dispersion parameters $\left(\hat{\alpha }\right)$ in leave-one-out-cross-validation (LOOCV) across device combinations (d0: hitachi-aloka noblus, d1: philips EPIQ 7 180 plus, d2: sonoSite titan). RMSLE: Root mean squared log error. *ρ*: Spearman's rank correlation coefficient. Scatter plot line is identity line. Bland-Altman plot lines are mean ± 1.96 standard deviations (SD) of $\ln(\hat{\alpha }/\alpha )$. Box plots demonstrate differential agreement by muscle site. UT, Upper trapezius; PM, Pectoralis major; MD, Middle deltoid; BR, Brachioradialis; RF, Rectus femoris; TA, Tibialis anterior.

Figures 3, 4 present scatterplots, Bland-Altman plots, and box plots visualizing agreement between predicted and measured µ and *α* values, with predictions obtained through LOOCV. The use of d1 as the outcome device consistently resulted in the best overall conversion model performance compared to d0 and d2. When predicted values were transformed from exponentiated values to raw values, this revealed substantial differences in the magnitude of prediction errors as predicted grayscale values increased. This observation was pronounced when predicting α. Differential bias by muscle site was not statistically significantly different than zero across all the conversion models for both *μ* and α.

## Discussion

Quantitative musculoskeletal ultrasound holds promise for objective assessment of muscle health, but its clinical adoption has been hindered by the lack of standardization across different ultrasound devices. Zero-inflated generalized negative binomial regression analysis of grayscale histograms revealed substantial inter-device variability in both muscle echogenicity (mean grayscale value, µ) and tissue heterogeneity (dispersion parameter, *α*) across three distinct ultrasound devices and six muscle sites. To address this, we developed device-to-device conversion models using heteroskedastic linear regression, successfully harmonizing µ across devices. While conversion of *α* also demonstrated adequate performance, we observed increased prediction errors at the extremes of predicted values, suggesting a need for further refinement. Our findings extend existing methods to standardize quantitative musculoskeletal ultrasound measurements across devices, with potential implications for clinical practice and research.

This work directly builds upon and extends our prior innovative approach to computational modeling of musculoskeletal ultrasound images. In our 2019 study (21), we first demonstrated the utility of fitting statistical distributions, including negative binomial regression, to grayscale histograms of muscle ultrasound images to quantify tissue heterogeneity beyond simple mean echogenicity. Specifically, the negative binomial dispersion parameter (*α*) and gamma shape parameter provided robust characterizations of histogram shape and overdispersion, showing stronger associations with peak grip strength in older adults (adjusted R² up to 0.70) compared to traditional mean grayscale values. That work established the clinical relevance of dispersion-based metrics for capturing age-related changes in muscle tissue composition estimates, particularly in community-dwelling older adults. However, that study was conducted within a single-device framework and did not address inter-device comparability. The current work advances this foundation by incorporating zero-inflation to handle device-specific artifacts (e.g., excess zeros in lower-dynamic-range systems) and by developing explicit heteroskedastic conversion models to harmonize both µ and *α* across three commercial ultrasound devices. This progression facilitates the development of a standardized approach to obtaining heterogeneity estimates for multi-device applications. Advancing this image analysis method would also overcome a major barrier to obtaining consistent ultrasound muscle composition estimates derived from echogenicity data.

Conversion performance varied across devices, which may be due to differences in ultrasound device-specific parameters associated with differences in transducer-system integration and proprietary attenuation compensation algorithms (e.g., time gain compensation or automated gain optimization). Our results showed the best conversion performance, particularly for *α*, when d1 (Philips EPIQ) was the outcome device. This may be due to a broader or more stable grayscale intensity distribution in this device. Conversely, d2 (SonoSite Titan) produced images with markedly lower µ, higher *α*, and greater zero inflation. These features likely reflect truncation at the lower end of the grayscale histogram, possibly due to lower default gain, narrower dynamic range, or device-specific image-processing behavior. These distribution patterns made modeling and conversion more difficult for this device. Encouragingly, our sensitivity analyses found that conversion performance did not differ by muscle site. This suggests our approach is robust to inherent differences in muscle architecture (e.g., pennation angle and fascial content).

Our findings align with and extend prior research on inter-device variability in musculoskeletal ultrasound grayscale analysis, while highlighting the advantages of our post-processing statistical harmonization approach over competing methods. Pillen et al. (12), compared quantitative skeletal muscle grayscale values between two ultrasound devices and found significant differences in mean echogenicity, attributing variability to manufacturer-specific processing algorithms. However, their study focused on direct comparison without proposing harmonization models, limiting its utility for multi-device applications. Similarly, Zaidman et al. (13), demonstrated that calibrated muscle backscatter measurements reduced inter-device variability compared to uncalibrated grayscale values, achieving reliable estimates across devices by accessing raw radiofrequency data for spectral processing and attenuation compensation. While methodologically sound, this backscatter approach requires devices that allow for raw data access, advanced calibration phantoms, and computational resources not readily available on most clinical systems, contrasting with our method's reliance on standard B-mode DICOM images for retrospective harmonization. More recently, Steffel et al., (22) examined the influence of ultrasound systems and gain on grayscale median (GSM) values using a phantom and human subjects, finding that gain adjustments (±10 dB/%) led to significant intra-system variability on at least 4 of 7 systems (*p* < 0.05) and inter-system differences across 5 of 7 devices (*p* < 0.05), and stressed the importance of standardizing device parameters and monitoring gain to mitigate system- and operator-dependent effects. Our work complements these findings by applying heteroskedastic regression to model both mean and variance in histogram parameters, achieving high agreement (RMSLE as low as 0.097 for µ) without gain recalibration during acquisition. These comparative studies describe variability without solutions, require pre-acquisition adjustments, or necessitate access to raw data. In contrast, our parametric modeling offers a viable pathway for harmonizing existing datasets, though it harmonizes processed outputs rather than raw signals.

The histogram-based modeling approach we present offers practical advantages over other standardization techniques. Methods based on quantitative backscatter analysis, for example, require access to raw radiofrequency data to measure tissue properties independent of device processing, as well as advanced data analytic methods for spectral processing, calibration, and attenuation compensation (13). These steps can introduce significant computational complexity and variability in multi-device or multi-center studies. However, radiofrequency data is often proprietary and inaccessible on most commercial clinical systems. Our method, in contrast, operates on the B-mode (DICOM) images themselves, making it applicable retrospectively and to a wider range of clinical devices. A consequence of this post-processing approach is that it requires the harmonization of the output of each device's proprietary image processing. This constraint highlights the importance of standardized acquisition settings when applying this conversion process, as our models were built using factory defaults. The applicability of the proposed method to images acquired with different settings (e.g., altered gain or time-gain compensation) remains unknown.

## 5 Limitations

This study has several limitations. First, our sample was small and demographically homogenous, consisting of nineteen older male U.S. Veterans who were generally overweight or obese. This limits the generalizability of our specific conversion models to women, other age groups, or populations with different health statuses and body compositions. This population may also have influenced the grayscale histogram distributions themselves. Older age is commonly associated with increased muscle echogenicity, while higher adiposity may increase subcutaneous tissue thickness and alter ultrasound attenuation before the beam reaches the target muscle (23). Therefore, the observed histogram parameters and derived conversion models may partly reflect the tissue characteristics and imaging challenges of an older, overweight/obese male population, and may not generalize directly to younger, leaner, female, or more diverse populations. Future studies should validate and, if necessary, recalibrate these conversion models in larger and more diverse cohorts, including women, younger and middle-aged adults, healthy controls, and patients with neuromuscular or other muscle-related disorders. Next, while we used LOOCV to assess model performance, we did not have a separate external validation cohort or a multi-operator validation dataset. Therefore, the true predictive accuracy and robustness of these models in new participants, different imaging environments, and real-world clinical practice have yet to be confirmed. Third, our conversion models are specific to the three device pairs tested. While they cannot be applied to other ultrasound systems without re-developing new, device-specific conversion parameters, the approach presented in this work does provide a promising approach to ultrasound image analysis across differing devices. Nonetheless, this limitation highlights the need for a more universal standardization approach, perhaps by calibrating all devices to a single reference standard or phantom. Although participants with conditions resulting in edema were excluded, we did not exclude participants solely because of subcutaneous fat thickness or reduced muscle quality, as these characteristics are common in the population studied and may be reflected in the grayscale histogram itself (24). However, this may limit applicability in cases where tissue depth or image artifact prevents reliable visualization of the target muscle or fascial borders. Moreover, our method's reliance on standardized image acquisition using factory default settings is a significant practical limitation. The imaging frequency was automatically governed by the effective center frequency inherent to the preset musculoskeletal scanning mode of each ultrasound device. It is also important to note that the robustness of these conversion models to any operator-controlled variations in settings, such as gain, time-gain compensation, or dynamic range, is unknown and a critical area for future investigation. Although images with substantial artifact or inadequate visualization were excluded, residual ultrasound artifacts such as acoustic shadowing, reverberation, anisotropy, or attenuation-related signal loss were not explicitly modeled or corrected.

## 6 Conclusions

We have demonstrated the feasibility of using a statistical modelling approach to harmonize quantitative grayscale histogram parameters across different clinical ultrasound devices. Our method harmonized mean echogenicity (µ, mean grayscale value) and showed promise for harmonizing tissue heterogeneity (*α*, dispersion parameter). This approach provides a viable, post-processing pathway for standardizing musculoskeletal diagnostic ultrasound data without significant muscle-site differential bias. Future work should focus on validating these models in larger, more diverse cohorts and exploring solutions for a more universal, setting-independent calibration.

## Data Availability

The datasets presented in this article are not readily available due to U.S. Department of Veterans Affairs (VA) policies governing human subjects research and the protection of Veterans' private and sensitive information. Consistent with VA Office of Research and Development requirements, data that include Protected Health Information (PHI) or Personally Identifiable Information (PII) cannot be shared outside the VA except as allowed under applicable Federal regulations, VHA privacy policies, and approved Data Use Agreements. Requests for data access may be directed to the corresponding author and will be evaluated in accordance with VA regulations, including privacy, security, and IRB requirements; however, data sharing outside the VA is typically not permitted for studies involving identifiable Veteran data. Requests to access the datasets should be directed to Michael Harris-Love, michael.harris-love@cuanschutz.edu.
